# The Abnormal Expression of B7‐H4 Is Associated With the Pathogenesis of Autoimmune Thyroid Diseases

**DOI:** 10.1155/jimr/5529891

**Published:** 2026-02-13

**Authors:** Yuqing Wu, Jianbin Xu, TianTian Cai, Yudie Fang, Zhaowei Huang, Xinwei Zhang, Guangxin Li, Wenyu Xu, Jinan Zhang

**Affiliations:** ^1^ Graduate School, Shanghai University of Traditional Chinese Medicine, Shanghai, 201203, China, shutcm.edu.cn; ^2^ Department of Endocrinology and Rheumatology, Shanghai University of Medicine & Health Sciences Affiliated Zhoupu Hospital, Shanghai, 201318, China, sumhs.edu.cn; ^3^ Shanghai University of Traditional Chinese Medicine, Shanghai, 201203, China, shutcm.edu.cn

**Keywords:** abnormality, autoimmune thyroid disease, B7-H4, expression

## Abstract

**Background:**

B7‐H4 is an immunosuppressive molecule extensively studied in tumor diseases and is also of interest in some autoimmune diseases. However, the relationship between B7‐H4 and autoimmune thyroid diseases (AITDs) has not been explored.

**Objective:**

To investigate the B7‐H4 expression in different tissues of patients with different subtypes of AITDs.

**Methods:**

The B7‐H4 protein expression in thyroid tissue of the participants was identified through immunohistochemical analyses while concentrations of plasma soluble B7‐H4 (sB7‐H4) were detected by enzyme‐linked immunosorbent assay (ELISA). Additionally, B7‐H4 mRNA expression in peripheral blood mononuclear cells (PBMCs) was evaluated via RT‐PCR.

**Results:**

The immunohistochemical findings revealed a decrease in levels of B7‐H4 protein in thyroid tissue of Graves’ disease (GD) and Hashimoto’s thyroiditis (HT) patients, compared with those of the normal controls. Similarly, a decrease was observed in the level of B7‐H4mRNA expression in PBMCs assayed via RT‐PCR. However, based on ELISA results, plasma levels of sB7‐H4 were increased in both GD and HT patients compared to those in normal controls.

**Conclusion:**

The abnormal expression of B7‐H4 in AITD patients suggests that it may be involved in the onset and progression of the disease. At the same time, the mechanism of action of B7‐H4 in AITD and whether it is a potential therapeutic target need to be further studied.

## 1. Introduction

B7‐H4, a type I transmembrane protein of the B7 family, is referred to as V‐set domain containing T‐cell activation inhibitor 1 (VTCN1) or B7S1, B7x [1]. At the same time, it is also an immunosuppressive molecule, with B7‐H4mRNA widely distributed in the peripheral tissues of healthy individuals [2]. However, its translation is strictly controlled, and B7‐H4 protein expression on the cell surface is restricted [2, 3]. Abnormal expression in multiple human cancer tissue types is related to advanced disease states, unfavorable cancer prognosis, as well as diminished patient survival rates [4]. Additionally, B7‐H4 has been observed to be abnormally expressed in inflammatory and autoimmune diseases [3, 5]. Currently, the B7‐H4 receptor located on the T cell surface has not yet been identified. However, studies have demonstrated that B7‐H4 is capable of binding to T cells, negatively regulates immune response reactions of T cells by inhibiting T cell proliferation, cell cycle, as well as cytokine secretion, and can promote immune escape [3].

Autoimmune thyroid diseases (AITDs) are prevalent autoimmune disorders that target specific organs. Graves’ disease (GD) and Hashimoto’s thyroiditis (HT) are two common types. Patients with these two disorders have a significant amount of lymphocyte infiltration in the thyroid tissue, and both disorders have the production of specific antibodies. GD is responsible for the vast majority of hyperthyroidism cases, accounting for about 90%; conversely, HT mainly causes hypothyroidism [6]. It is currently believed that the pathogenesis of AITDs is synergistically influenced by genetic, epigenetic, and environmental factors as well as the key role of the loss of immune tolerance towards thyroid self‐antigens [7].

As an immune negative regulatory molecule, B7‐H4 is related to immune response. There have been studies on the relationship between B7‐H4 and autoimmune diseases, which include systemic lupus erythematosus (SLE) [8] and rheumatoid arthritis (RA) [9], but there is no detailed literature report on B7‐H4 and AITDs. Therefore, this study focuses on the correlation between AITDs and B7‐H4, including its expression and effects.

## 2. Methods and Materials

### 2.1. Clinical Characteristics of Participants

In the current study, 55 patients diagnosed with GD and 29 patients diagnosed with HT were recruited from the Department of Endocrinology, Shanghai University of Medicine & Health Sciences Affiliated Zhoupu Hospital. Additionally, 40 normal controls of matched gender and age were selected from the Physical Examination Center of this hospital (excluding immunohistochemical subjects). AITD was determined through a comprehensive evaluation that considers clinical manifestations, laboratory tests, and imaging examinations [10]. The inclusion criteria of GD were hyperthyroidism, diffuse goiter, decreased thyroid stimulating hormone (TSH), and positive TSH receptor antibody (TRAb). The inclusion criteria for HT consisted of elevated levels of anti‐thyroglobulin antibody (TGAb) or anti‐thyroid peroxidase antibody (TPOAb), goiter, with or without hypothyroidism. Screening of normal controls excluded disease‐related clinical symptoms, positive TPOAb, and other abnormalities. Serum levels of TPOAb, TGAb, TSH, free triiodothyronine (FT3), and free thyroxine (FT4) were detected by electrochemiluminescence (Roche, Basel, Switzerland). The patients included in the study did not have any other chronic or autoimmune diseases. The clinical research characteristics of the participants are displayed in Table 1, with each trial having a different number of participants, as outlined in Table S1. Details of immunohistochemistry (IHC) are provided in Table S2. Our research was conducted in compliance with the Declaration of Helsinki, with ethical approval from the Ethics Committee of Zhoupu Hospital (2023‐C‐123‐E01); all the participants in the present study provided informed consent.

**Table 1:** The clinical characteristics of the study subjects.

Variables	NC	GD	HT	*p*‐Value (ANOVA)
Total (N)	40	55	29	—
Age (years)	36.45 ± 10.40	36.05 ± 13.37	34.76 ± 10.51	0.71
Gender (F/M)	23/17	37/18	20/9	0.52
FT3 (pmol/L)	—	21.29 (13.50,30.72)	5.15 (4.51,5.67)	—
FT4 (pmol/L)	—	37.73 (29.81,49.16)	12.82 (12.22,15.70)	—
TSH (μIU/mL)	—	0.001 (0.001, 0.005)	1.760 (0.634, 2.784)	—
TRAb (IU/L)	—	11.58 (5.51,20.71)	—	—
TPOAb (IU/mL)	—	326.50 (36.73,707.11)	468.75 (113.56,1000.00)	—

*Note:* Reference range: FT3, 2.62–5.69 pmol/L; FT4, 9.01–19.05 pmol/L; TSH, 0.350–4.940 μIU/mL; TGAb <4.11 IU/mL; TPOAb <5.61 IU/mL; TRAb <1.50 IU/L.

Abbreviations: F, female, GD, Graves’ disease; HT, Hashimoto’s thyroiditis; M, male; NC, normal control.

### 2.2. Isolation of Peripheral Blood Mononuclear Cells (PBMCs)

Venous blood of subjects was collected using EDTA anticoagulation tubes and centrifuged; sufficient supernatant was taken as plasma sample and frozen at −80°C for subsequent soluble B7‐H4 (sB7‐H4) detection. PBMCs were then isolated separately from the remaining blood samples by adopting the Ficoll density gradient method and following the instructions for the production of lymphocyte isolation medium (DAKEWE, Beijing, China).

### 2.3. IHC for B7‐H4 in Thyroid Tissues

The thyroid tissues from four patients with GD and four patients with HT were selected for immunohistochemical detection, and compared with three normal thyroid tissues. Normal thyroid tissue was derived from the extratumoral tissue of a benign adenoma of the thyroid gland that had been unilaterally resected. Thyroid tissues were embedded in paraffin, fixed, made into sectioned, removed, and antigenically repaired with citrate buffer (PH 6.0). After endogenous oxidase was blocked, serum was then blocked before incubating with a primary antibody (anti‐VTCN1, rabbit, 1:1200, Proteintech, Wuhan, China, 12080‐1‐AP) overnight. HRP‐labeled secondary antibody (Jackson ImmunoResearch Laboratories, West Grove, USA, 1:1200) was added for staining and incubated for 30 min, then DAB color solution was added for color development and then counterstained with hematoxylin. After that, the slices were dehydrated, sealed, and photographed under the microscope.

### 2.4. Quantitative Real‐Time Polymerase Chain Reaction (qRT‐PCR) for B7‐H4 mRNA Expression in PBMCs

The obtained PBMCs were lysed by Trizol reagent (TakaRa, Dalian, China) to release the intracellular RNA. This cell lysate was mixed with chloroform to separate the RNA from the water phase and then underwent precipitation, drying, and redissolving to obtain the RNA solution. The cDNA was reverse transcribed with 1 μg of RNA according to the instructions of the PrimeScript RT kit (TaKaRa, Dalian, China). Target genes in the cDNA of participants were detected by qRT‐PCR using the SYBR Premix Ex TaqTM II (TaKaRa, Dalian, China) reagent utilizing an ABI PRISM 7500, with β‐actin and B7‐H4 as primer sequences. The relative expression of each gene was analyzed by the method of 2^−△△C^, and used β‐actin as an internal control. Primers of PCR used were as follows: B7‐H4 (forward: CACCAGGATAACATCTCTCAGTGAA, reverse: TGGCTTGCAGGGTAGAATGA), β‐actin (forward: CATTGCCGACAGGATGCAG, reverse: CTCGTCATACTCCTGCTTGCTG).

### 2.5. Enzyme‐Linked Immunosorbent Assay (ELISA) for Plasma sB7‐H4 Levels

The plasma sB7‐H4 levels were measured through double antibody sandwich ELISA using a VTCN1‐containing detection kit (Cloud Clone Wuhan, China). The samples were derived from the supernatant plasma obtained from blood collected in tubes following centrifugation at 2000 rpm for 10 min, and the detection was conducted following the provided instructions.

### 2.6. Statistical Analysis

Continuous data are presented as mean ± standard deviation, while the other data with non‐normal distribution are reported as median (25^th^–75^th^ percentile). In parametric tests, independent samples between two groups were compared using Student’s *t*‐test, and other comparisons of independent samples between multiple groups were conducted by the method of one‐way ANOVA, with multiple pairwise comparisons performed. However, the values within the groups did not conform to normal distribution or variance. The Mann–Whitney *U* test compared independent samples between two groups, while the Kruskal–Wallis test compared independent samples between multiple groups. Additionally, a multiple pairwise comparison test was conducted. A significant difference was determined if *p* < 0.05. All data were analyzed by the software of GraphPad Prism 9.0.

## 3. Results

### 3.1. Elevation of B7‐H4 Expression Levels in the GD and HT Patients

The immunohistochemical results indicated positive B7‐H4 protein expression in thyroid tissue, with the presence of brown staining. As shown in Figure 1A, some brown particles could be seen in the section of the control group, mainly located on the membrane of thyroid follicular cells. There were fewer brown particles in thyroid follicular epithelial cells and lymphocytes in the GD and HT groups, and the staining was not obvious. For quantification, three pictures were randomly intercepted from each sample for analysis. Figure 1B uses the average integrated optical density (IOD) as the image analysis measure. Compared with the normal controls, a decrease was found in the B7‐H4 protein expression of the GD and HT patients. At the same time, there was a statistical difference between the normal controls and the HT patients (*p* < 0.01). In contrast, Figure 1C shows the proportion of B7‐H4 staining areas in groups. The GD and HT groups showed a significant reduction in positive areas of thyroid when compared to the normal control group (*p* < 0.05; *p* < 0.01).

Figure 1B7‐H4 levels in thyroid tissue of the GD and HT patients. (A) Immunohistochemical results of B7‐H4 in thyroid tissue specimens. (B) Statistical analysis of mean optical density after B7‐H4 immunohistochemical staining. (C) Comparison of B7‐H4 protein positive area (%).  ^∗∗^ *p* < 0.01,  ^∗^ *p* < 0.05. NC = 3, GD = 4, HT = 4. GD, Graves’ disease; HT, Hashimoto’s thyroiditis; NC, normal control.

**Figure:**
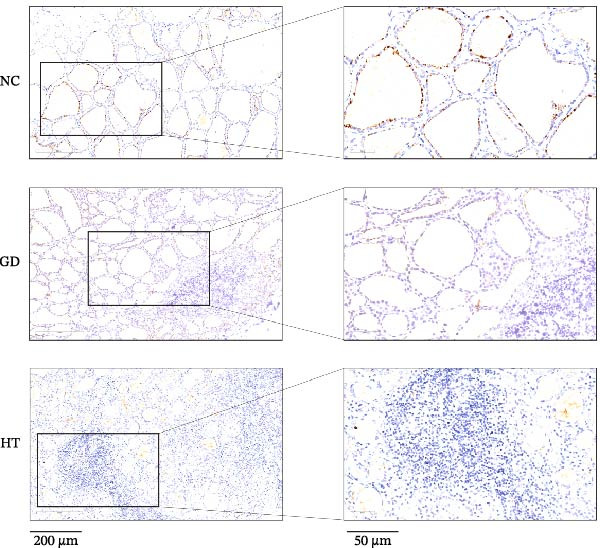
(A)

**Figure:**
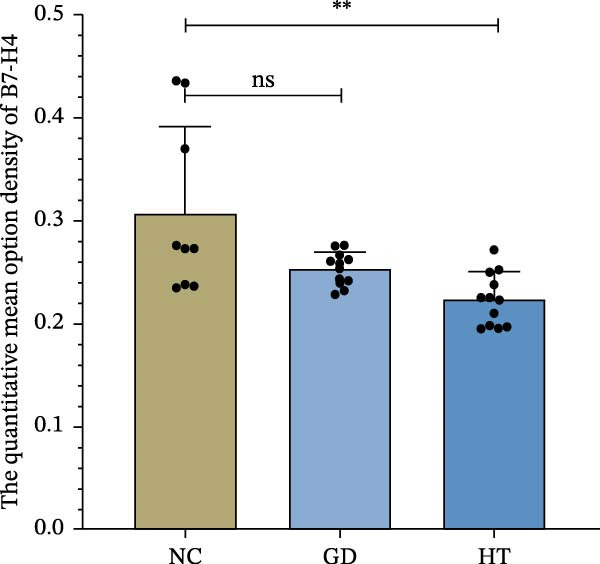
(B)

**Figure:**
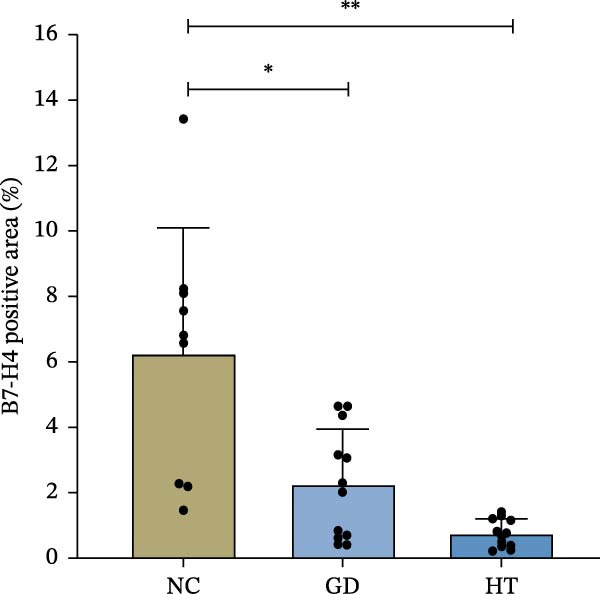
(C)

### 3.2. Decrease of B7‐H4 mRNA Expressions in Both the GD and HT Patients

The expression of B7‐H4 mRNA in PBMC was detected by qPCR. Figure 2A shows the expression levels of B7‐H4 mRNA in each group. Figure 2B,C present the scatter diagram, which depicts the distinct numerical disparities between the normal controls and patients. The results revealed a significant decrease in B7‐H4 mRNA expression of the GD and HT patients compared to the normal controls (*p* < 0.05). Nevertheless, there was no statistical difference found between GD and HT.

Figure 2B7‐H4 mRNA expression levels in the GD and HT patients. (A) Statistical analysis of B7‐H4 mRNA expression levels in the GD and HT patients ( ^∗^ *p* < 0.05). NC = 25, GD = 30, HT = 25. (B) Difference of B7‐H4 mRNA levels between GD and NC. (C) Difference of B7‐H4 mRNA levels between HT and NC. GD, Graves’ disease; HT, Hashimoto’s thyroiditis; NC, normal control.

**Figure:**
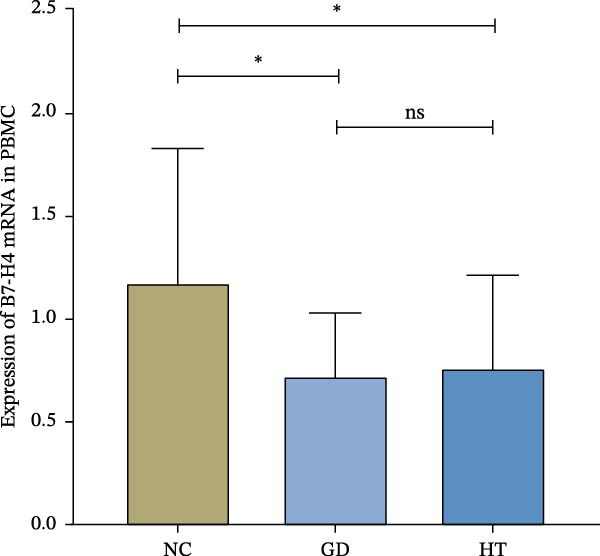
(A)

**Figure:**
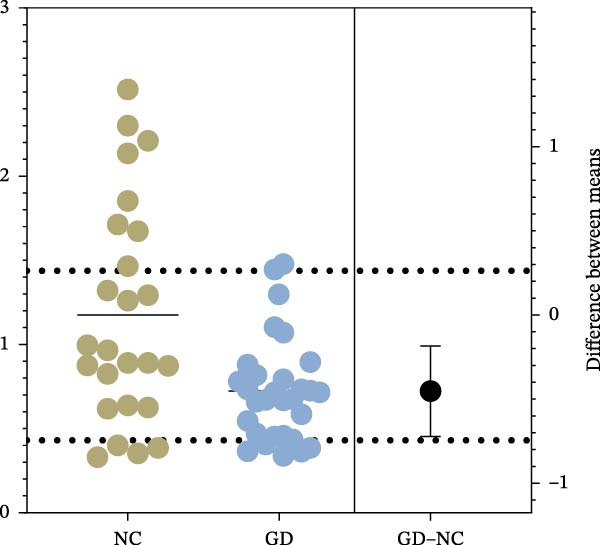
(B)

**Figure:**
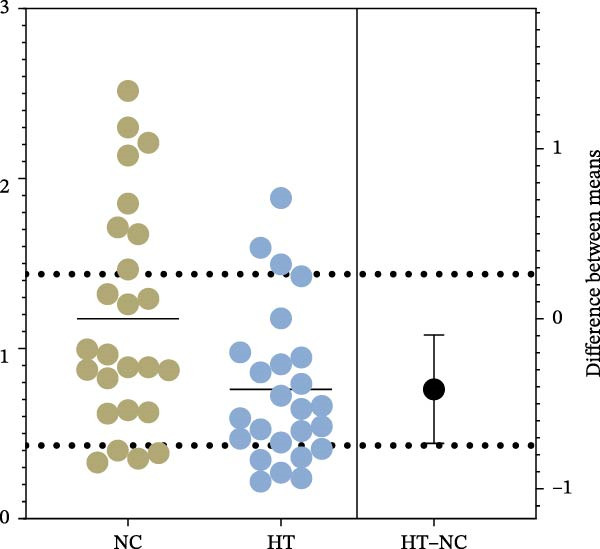
(C)

### 3.3. Increase of Plasma Concentrations of sB7‐H4 in the GD and HT Patients

The concentrations of plasma sB7‐H4 were detected by ELISA. As shown in Figure 3A, the violin chart can intuitively reveal the distribution characteristics and similarity of concentrations between different groups and can show the significance of differences. The scatter diagram can show the specific numerical differences between the normal individuals and patients (Figure 3B,C). Compared with the normal control group, the concentrations of sB7‐H4 were significantly increased in both the GD and HT groups (*p* < 0.001; *p* < 0.0001), and the difference between the HT and normal control groups was more significant. Additionally, sB7‐H4 was also elevated significantly in HT plasma compared to GD (*p* < 0.001).

Figure 3Plasma concentration of sB7‐H4 in the GD and HT patients. (A) Violin plot of the difference in plasma sB7‐H4 concentration between GD and HT patients (NC = 25, GD = 30, HT = 22).  ^∗∗∗∗^ *p* < 0.0001,  ^∗∗∗^ *p* < 0.001. (B) Difference of plasma sB7‐H4 concentration between GD and NC. (C) Difference of plasma sB7‐H4 concentration between HT and NC. GD, Graves’ disease; HT, Hashimoto’s thyroiditis; NC, normal control.

**Figure:**
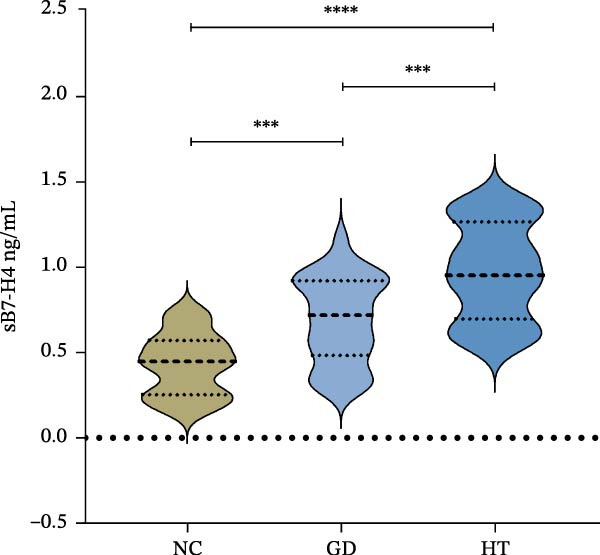
(A)

**Figure:**
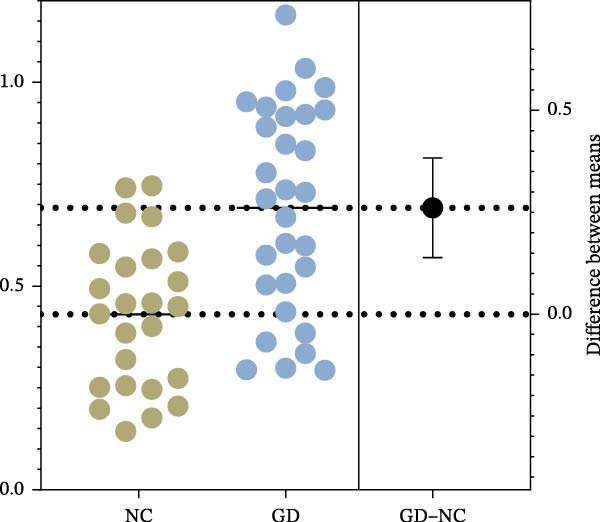
(B)

**Figure:**
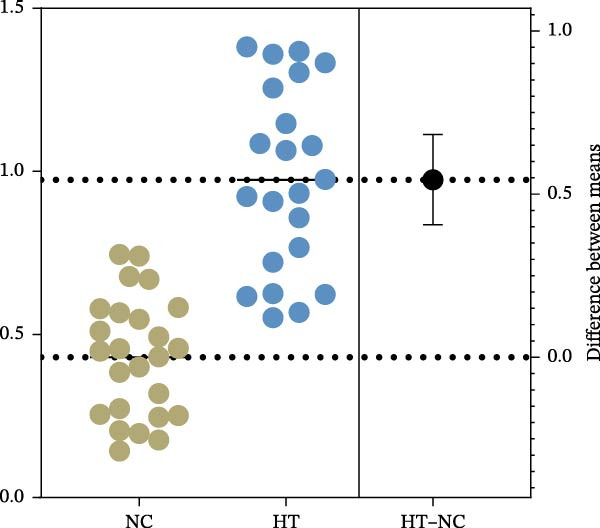
(C)

### 3.4. Relationship Between B7‐H4 Expression and Clinical Indicators in the GD Patients

We further analyzed the correlation between clinical indicators and the expression level of B7‐H4 in the GD patients. As shown in Figure 4, FT4 level in the patients was significantly negatively correlated with sB7‐H4 concentration, while other indicators were not correlated.

Figure 4Correlation between B7‐H4 mRNA expression levels and clinical indicators in GD. (A) Correlation between FT4 and B7‐H4 mRNA levels in the GD patients. (B) Correlation between TRAb and B7‐H4 mRNA levels in the GD patients. (C) Correlation between FT4 and plasma sB7‐H4 concentration in the GD patients. (D) Correlation between TRAb and plasma sB7‐H4 concentration in the GD patients.

**Figure:**
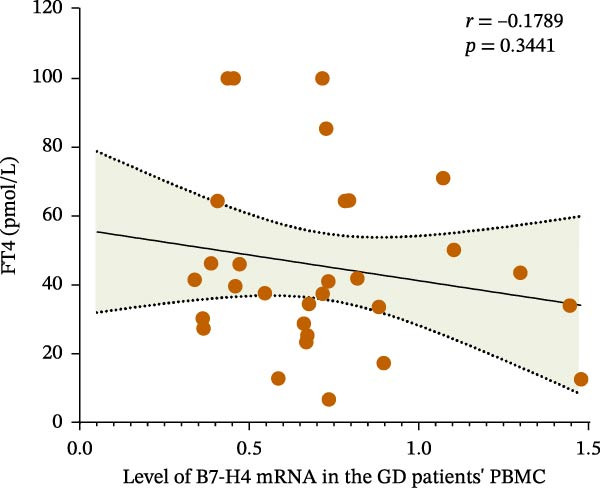
(A)

**Figure:**
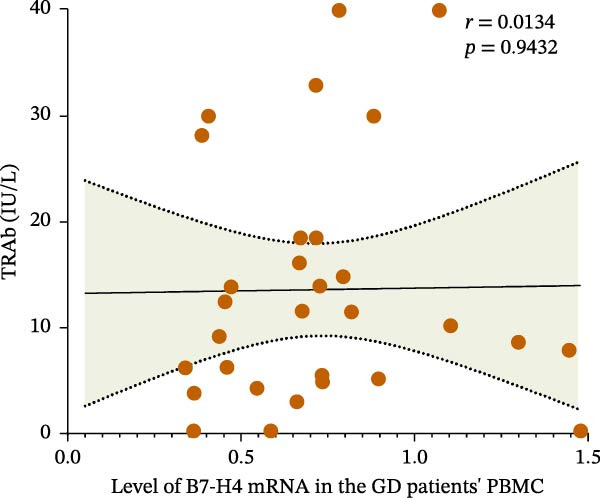
(B)

**Figure:**
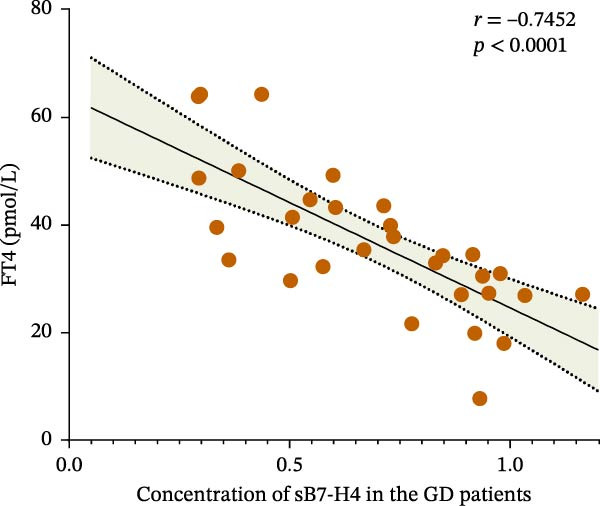
(C)

**Figure:**
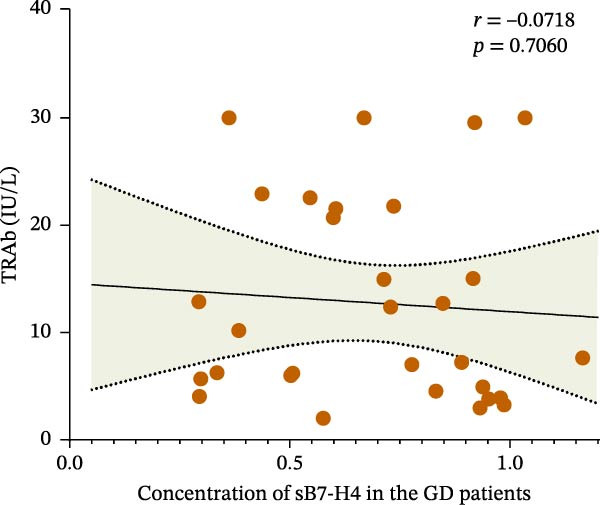
(D)

## 4. Discussion

B7‐H4 has emerged as a possible therapeutic target for a range of medical conditions, including tumors, inflammation, autoimmune diseases, as well as organ transplantation [1, 3, 11]. As an important B7 family member, it regulates T cell function in inflammation, thereby mediating multiple immunosuppressive mechanisms [12]. B7‐H4 can suppress the inflammatory function of effector T cells by decreasing the production of cytokines like IFN‐γ and by inhibiting proliferation [2, 13]. On the other hand, it can enhance the functions of Treg cells, which include the secretion of immunosuppressive factors like IL‐10 [14, 15]. In recent years, the role of B7‐H4 in tumorigenesis has been extensively researched. Numerous studies demonstrate that B7‐H4 is overexpressed in both tumor cells and tumor‐associated macrophages, leading to the facilitation of immune escape by the tumor [3, 16]. These studies have investigated its overexpression in various cancers such as gastric cancer [17], lung cancer [18], and ovarian cancer [19]. The degree of malignancy, stage of the tumor, and poor prognosis are related to the level of B7‐H4 expression [20]. Therefore, it has become a potential target for tumor immunotherapy [16, 21]. Current tumor therapies targeting B7‐H4 include monoclonal antibodies, double antibodies, ADCs, and cell therapy [22, 23].

The role of B7‐H4 in multiple autoimmune diseases has attracted much attention [24]. Although previous studies have demonstrated that B7‐H4 is essentially undetectable in normal tissues, our findings indicate that B7‐H4 is expressed at a low level in thyroid follicular epithelial cells of healthy individuals, which is consistent with the data presented on the Protein Atlas website (https://www.proteinatlas.org/). The proportion of positive area and the staining intensity were statistically analyzed in the results of IHC. The B7‐H4 expression was lower in the thyroid tissues of the GD and HT patients compared to the normal subjects. In addition, our qPCR results also showed a decrease in B7‐H4 mRNA expression in PBMCs from the GD and HT patients compared to the normal controls. These above results indicated that a deficiency in B7‐H4 expression was identified in AITDs. Therefore, we speculate that the B7‐H4 loss could lead to the decreased inhibition of T cell activation and immune homeostasis imbalance, which is hypothesized to be a potential mechanism of AITDs. Previous studies on other autoimmune diseases have provided indirect support for our hypothesis. Research has shown that primary Sjögren’s syndrome patients lack B7‐H4 expression in salivary glands and peripheral blood, which impairs the regulatory function of T cells [25]. Furthermore, B7‐H4 expression is reduced in pancreatic islet β‐cells, accompanied by insulin expression but not glucagon expression, which plays a critical role in maintaining β‐cell function [26]. Depletion of B7‐H4 aggravates lymphoid tissue swelling and renal lesions in a mouse model of SLE, while B7‐H4‐Ig infusion attenuates the manifestations of lupus [27].

Contrary to the results of IHC and qPCR, results of ELISA revealed that the concentration of plasma sB7‐H4 in the GD and HT patients was significantly increased than that in normal controls. In addition, there was also a significant difference between GD and HT. This was a departure from our original assumption. The study by Ding et al. [28] also found that serum sB7‐H4 levels were significantly increased in patients with autoimmune diseases, including GD patients. We have discussed and considered the reason why B7‐H4 does not fall but rises. Studies suggest that the loss of B7‐H4 in islet β cells as well as antigen‐presenting cells (APCs) is related to immune‐mediated β‐cell death and the subsequent onset of autoimmune diabetes [26, 29]. B7‐H4 protein loses its transmembrane segment on the surface of APC cells in islets due to proteolytic cleavage of metalloproteinase lipolysin, and is released in soluble form [29]. Therefore, we infer that in AITDs, B7‐H4 on the membrane is cleaved and released in a soluble form, which reduces its expression on the cell surface and increased expression in plasma.

sB7‐H4 is elevated in ovarian, breast, gastric, lung, and liver cancers, potentially serving as a marker for predicting cancer prognosis and progression [30]. In the field of autoimmune diseases, elevated levels of sB7‐H4 have been observed early in the progression of type 1 diabetes mellitus (T1DM) and are linked to the rate of disease progression [26, 29]. In RA patients, elevated sB7‐H4 levels are found to be associated with disease activity as well as higher levels of serum C‐reactive protein, which can be a nonspecific surrogate marker for persistent inflammation [31]. Experimental studies in mice showed that sB7‐H4 can hinder the inhibitory function of B7‐H4 present on the cell surface, thus exacerbating collagen‐induced arthritis and promoting the progression of diseases, and serving as a decoy molecule [31]. In this study, only a correlation was found between FT4 levels and sB7‐H4 in GD, while no significant correlation was found between B7‐H4 and other clinical indicators. However, the study found compared to that in GD patients, the concentration of sB7‐H4 was higher in HT patients. We speculate that the higher sB7‐H4 concentration in HT might be because B7‐H4 is related to the degree of inflammation. After all, compared to the GD ones, the HT patients exhibit higher levels of lymphocyte infiltration in the thyroid gland. In HT, the thyroid gland is subjected to autoimmune attack, and T and B cells infiltrate into the thyroid tissue, leading to the accumulation and destruction of inflammatory cells [32]. The inflammatory reaction of GD is relatively mild, and the lesions are mainly located in thyroid follicular epithelial cells [33].

## 5. Conclusion

Based on the findings of our study, we uncovered that B7‐H4 expression is abnormal in GD and HT for the first time, which suggests that this protein may be linked to the development and onset of AITDs. Further studies are also needed to explore the potential of B7‐H4 in the treatment of AITDs.

## Author Contributions

**Yuqing Wu**: conceptualization, visualization, investigation, formal analysis, data curation, writing – original draft, writing – review and editing. **Jianbin Xu**: methodology, software, visualization. **TianTian Cai**: methodology, formal analysis, visualization, writing – review and editing. **Yudie Fang**: software, validation. **Zhaowei Huang**: conceptualization, methodology, writing – review and editing. **Xinwei Zhang**: data curation, supervision. **Guangxin Li and Wenyu Xu**: methodology, supervision. **Jinan Zhang**: conceptualization, funding acquisition, resources, supervision, project administration, writing – review and editing.

## Funding

This research was funded by the Project of the National Natural Science Foundation of China (Grant 82370791), the Shanghai Pudong New Area Science and Technology development Fund Public Institutions Livelihood Research Project (Grant PKJ2023A‐Y10), Key discipline project of Shanghai Pudong New Area Health Commission (Grant PWZxk2022‐22), and the Clinical Research Center of Thyroid Diseases of Shanghai Health Medical College (Grant 20MC20200002).

## Ethics Statement

The studies involving human participants were reviewed and approved by Ethics Committee of Zhoupu Hospital of Shanghai University of Medicine & Health Sciences.

## Consent

The patients/participants provided written informed consent to participate in this study.

## Conflicts of Interest

The authors declare no conflicts of interest.

## Supporting Information

Additional supporting information can be found online in the Supporting Information section.

## Supporting information


**Supporting Information** The file called Supporting Tables is the supporting table referred to in the text and includes Table S1 (demographics of participants) and Table S2 (the clinical characteristics of the immunohistochemistry subjects).

## Data Availability

The data that support the findings of this study are available from the corresponding author upon reasonable request.
