# Supplementary material for: The Abnormal Expression of B7‐H4 Is Associated With the Pathogenesis of Autoimmune Thyroid Diseases
Source: J Immunol Res. 2026 Feb 13;2026:5529891. doi: 10.1155/jimr/5529891 (PMC13140884; doi:10.1155/jimr/5529891)
Supplement: Supplementary file 1 — Supporting Information The file called Supporting Tables is the supporting table referred to in the text and includes Table S1 (demographics of participants) and Table S2 (the clinical characteristics of the immunohistochemistry subjects). [file JIMR-2026-5529891-s001.docx]

**Supplementary Table S1.** Demographics of participants.

| B7-H4mRNA by qPCR | Normal control | Grave's disease | Hashimoto thyroiditis |
| --- | --- | --- | --- |
| Participants (female /total n) | 16/25 | 19/30 | 18/25 |
| HT with hypothyroidism |  |  | 2/25 |
| Newly-diagnosed GD |  | 25/30 |  |
| Age(years) | 37.60±11.11 | 34.70±12.08 | 34.58±11.41 |
| ELISA of soluble B7-H4 | Normal control | Grave's disease | Hashimoto thyroiditis |
| Participants (female /total n) | 12/25 | 21/30 | 15/22 |
| HT with hypothyroidism |  |  | 2/22 |
| Newly-diagnosed GD |  | 28/30 |  |
| Age(years) | 36.16±9.83 | 36.17±14.10 | 34.59±12.10 |

**Supplementary Table S2.** The clinical characteristics of the immunohistochemistry subjects

|  | Age  (years) | Gender  (F/M) | FT3  (pmol/L) | FT4  (pmol/L) | TSH  (IU/mL) | TPOAb (IU/mL) | TGAb  (IU/mL) | TRAb  (IU/L) |
| --- | --- | --- | --- | --- | --- | --- | --- | --- |
| Normal control |  |  |  |  |  |  |  |  |
| NC1 | 45 | F | -Normal- | | | | | |
| NC2 | 45 | F | -Normal- | | | | | |
| NC3 | 33 | F | -Normal- | | | | | |
| Grave's disease |  |  |  |  |  |  |  |  |
| GD1 | 30 | F | 4.3 | 10.27 | 0.932 | 807.22 | 27.58 | / |
| GD2 | 40 | F | 3.35 | 13.81 | 9.890 | 45.40 | 10.00 | 24.71 |
| GD3 | 51 | F | 4.58 | 7.49 | 0.002 | >1000.00 | >1000.00 | 23.16 |
| GD4 | 32 | F | 7.54 | 18.07 | 0.001 | 320.44 | 20.00 | 7.19 |
| Hashimoto thyroiditis |  |  |  |  |  |  |  |  |
| HT1 | 38 | F | 3.43 | 10.33 | 43.05 | 44.70 | 694.00 | / |
| HT2 | 60 | F | 4.80 | 15.82 | 1.628 | 819.90 | 19.80 | / |
| HT3 | 56 | F | 4.30 | 14.86 | 1.634 | 36.90 | 83.10 | / |
| HT4 | 52 | F | 3.39 | 14.03 | 1.821 | 30.00 | 57.40 | / |

F: female, M: male; Reference range: FT3, 2.62-5.69 pmol/L; FT4, 9.01-19.05 pmol/L; TSH, 0.350-4.940 μIU/mL; TGAb <4.11 IU/mL; TPOAb <5.61 IU/mL; TRAb <1.50 IU/L.
